# A Photoelectrochemical Sensor Based on Anodic TiO_2_ for Glucose Determination

**DOI:** 10.3390/s19224981

**Published:** 2019-11-15

**Authors:** Karolina Syrek, Maciej Skolarczyk, Marta Zych, Monika Sołtys-Mróz, Grzegorz D. Sulka

**Affiliations:** Department of Physical Chemistry & Electrochemistry, Faculty of Chemistry, Jagiellonian University, Gronostajowa 2, 30387 Krakow, Poland

**Keywords:** titanium oxide, anodization, photoelectrochemical sensor, glucose oxidation

## Abstract

A simple photoelectrochemical (PEC) sensor based on non-modified nanostructured anodic TiO_2_ was fabricated and used for a rapid and sensitive detection of glucose. The anodic TiO_2_ layers were synthesized in an ethylene glycol-based solution containing NH_4_F (0.38 wt.%) and H_2_O (1.79 wt.%) via a three-step procedure carried out at the constant voltage of 40 V at 20 °C. At the applied potentials of 0.2, 0.5, and 1 V vs. saturated calomel electrode (SCE), the developed sensor exhibited a photoelectochemical response toward the oxidation of glucose, and two linear ranges in calibration plots were observed. The highest sensitivity of 0.237 µA µmol^−1^ cm^−2^ was estimated for the applied bias of 1 V. The lowest limit of detection (LOD) was obtained for the potential of 0.5 V vs. SCE (7.8 mM) with the fastest response at ~3 s. Moreover, the proposed PEC sensor exhibited relatively high sensibility, good reproducibility, and due to its self-cleaning properties, a good long-term stability. Interfering tests showed the selective response of the sensor in the presence of urea and uric acid. Real-life sample analyses were performed using an intravenous glucose solution, which confirmed the possibility of determining the concentration of analyte in such types of samples.

## 1. Introduction

Over the past decade, interest in glucose determination has risen significantly, not only because of the increasing number of people suffering from diabetes mellitus (one of the leading causes of human death [1]) but also for environmental and bioindustial process monitoring, or even quality control in the food industry [2,3,4]. Glucose determination can be performed using a recently proposed group of sensors that operate under sunlight and exploit the photoelectrochemical (PEC) properties of advanced semiconducting materials [5]. The growing interest in such devices arises from their unique properties, including a wide range of linear responses, inherent miniaturization, portability, and easy integration in existing applications [5]. A basic PEC system is composed of a semiconductor photoelectrode that absorbs photons with sufficient energy to excite electrons of an active species on it from the valence to the conduction band in order to generate electron-hole pairs. The excited electrons travel through the semiconductor layer and reach the counter electrode via the external circuit, while holes oxidize species at the semiconductor surface, and photocurrent response of the system is observed [6,7,8]. In addition, a big advantage of PEC sensors is a separation of the excitation source (light) and detection signal (photocurrent), which facilitates a low background signal, and secures a high sensitivity and fast sensor response [9,10,11,12]. Active centers of platinum-based electrochemical sensors are often blocked by glucose oxidation products [13]. In contrast, PEC sensors based on semiconductor materials, which often possess strong photocatalytic properties under irradiation conditions, can counteract the loss of active surface area of the electrode by oxidizing the impurities remaining on the electrode surface (so called self-cleaning properties) [14].

Anodic nanostructured metal oxides are very attractive electrode materials for the construction of sensing devices due to their excellent optical, electrical, and chemical properties that are strongly affected by their extraordinary morphology, which may be precisely designed, and controlled by synthesis conditions [15]. Among recently investigated photo- and electro-chemical sensors that are based on anodic metal oxides, anodic titanium dioxide (ATO) based sensors are the most commonly used forms [15,16] due to their good chemical stability, biocompatibility, non-toxicity, and well-known photocatalytic properties [17]. A variety of demonstrated PEC sensors is strictly related to ongoing studies aimed at designing of ATOs with enhanced properties, which can improve the detection limits of different compounds (e.g., glucose). Nowadays, modifications of ATO are focused on introducing into the anodic structure and/or depositing on its surface noble metals (Ag, Pt [10,17,18,19,20,21,22,23,24,25,26], transition metals (Co, Cu [27]), metal oxides (e.g., CuO, Cu_2_O [28,29,30]), and others. As the glucose oxidation path leads typically to gluconolactone and hydrogen peroxide, PEC sensors based on ATO layers have been developed for determination of glucose or H_2_O_2_ [31]. Selected examples of PEC glucose (GLU) sensors are summarized in Table 1.

From a practical point of view, a reliable sensor should detect the desired molecule (e.g., glucose) at very low concentrations with a high sensitivity and fast response. It is especially important not only for clinical detection and analysis of biological and environmental samples, but also for quality control in the food industry [3,4]. Reported in the literature [17,18] ATO-based PEC sensors for glucose determination are reportedly characterized by a sensitivity of 0.14 µA µmol^−1^ cm^−2^ with a linear response range from 0 to 1200 µmol dm^−3^ and detection limit of about 2.7 µmol. As can be seen (Table 1), these parameters can be significantly improved when additional noble metal nanoparticles are deposited on the oxide layer [18]. It should be emphasized that the PEC properties of nanostructured materials are strictly related to their morphology. For the sensing properties of ATO, the particularly important parameters are the thickness of the oxide layer and its porosity [32,33]. Therefore, the advantage of the ATO structure itself should be carefully explored, instead of modification of its surface with an additional component (e.g., metal nanoparticles) in the subsequent research stage. On the other hand, sensing characteristics of the sensor also depend on the environmental conditions in which the sensor is used. For instance, Feng et al. [18] investigated the influence of applied potential, light intensity, and electrolyte pH on the PEC sensing ability of glucose on ATO. At potentials within the range of 0.2–0.6 V vs. Ag/AgCl, similar sensitivities (0.112 and 0.115 µA µM^−1^) were observed, but at higher potentials, lower sensitivity was found. The optimal conditions for the effective PEC sensor were defined as the polarization potential of 0.2 V vs. Ag/AgCl, light intensity of 36 mW cm^−2^, and neutral pH. However, a relatively long time response of about 56 s was observed.

The selectivity of electrochemical sensors is also a very important issue. Essentially, the selective sensor should be able to respond to a certain reaction enabling the detection of a specific component. Typically, in real-life samples other electroactive interfering substances are also present. In non-enzymatic glucose detection, such compounds as ascorbic acid, uric acid, and lactic acid can simultaneously oxidize on the electrode surface. The sensitivity of electrodes can be improved by providing a large active area of the material [14], and for this reason, nanostructured electrodes are very attractive, however as was already mentioned, their surface can be blocked by semi-products.

Therefore, in this work we present, for the first time, a detailed and systematic study on PEC sensing properties of ATO toward glucose oxidation. An important part of this study was to evaluate an optimal wavelength and applied potential of glucose determination in order to provide a sensitive and stable performance of the sensor with a low limit of detection. For each of the tested potentials, the time response of the system was studied, taking into account the different kinetics of the observed photocurrent. The fabricated PEC sensor was used for the determination of glucose in the presence of interfering substances, and in real-life samples. The stability of obtained sensor was also tested, and PEC performance of the freshly prepared ATO was compared after one and three months of storage.

## 2. Materials and Methods

### 2.1. ATO Synthesis and Characterization

Prior to anodization, a titanium foil (99.5% in purity, 0.25 mm thick, Alfa Aesar) was polished electrochemically, and then chemically [32,34,35]. Nanostructured ATO layers were synthesized in an ethylene glycol-based solution containing NH_4_F (0.38 wt.%) and H_2_O (1.79 wt.%) in a three-step procedure carried out at a constant voltage of 40 V at 20 °C. The first and second anodizing steps lasted for 3 h. After each step, an adhesive tape was used to mechanically remove the obtained oxide layers. The third anodizing step was carried out for 10 min in a freshly prepared electrolyte. During each step, a constant stirring rate of 200 rpm was provided [36]. As-received amorphous materials were subjected to annealing in air at 400 °C for 2 h with a heating rate of 2 °C min^−1^ using a muffle furnace (FCF 5SHM Z, Czylok, Poland) [35]. The morphology of ATO layers was characterized using a field emission scanning electron microscope (SEM, Hitachi S-4700, Japan). The diffraction patterns of ATO were registered using X-ray diffractometer Rigaku Mini Flex II with a Cu Kα radiation (1.54060 Å) at the 2θ range of 20–60°. For the analysis of diffraction patterns, the International Centre for Diffraction Data (ICDD) database was used.

### 2.2. Photoelectrochemical Study

PEC measurements were performed in a 0.1 M KNO_3_ (pH = 6.9) solution at constant temperature (20 °C) using a three-electrode cell with a quartz window, where the nanostructured ATO layers were used as a working electrode, a platinum foil as a counter electrode, and a Luggin capillary with a saturated calomel electrode (SCE) as a reference electrode (caution should be exercised when using a mercury-containing electrode). The generated photocurrent was measured using a photoelectric spectrometer equipped with the 150 W xenon arc lamp and coupled with a potentiostat (Instytut Fotonowy, Poland) [32,33,37]. PEC characterization was performed at the potential range of 0–1 V over wavelengths ranging from 300 to 400 nm. For a given polarization potential, the photoanode was sequentially illuminated with the UV light (in the range of 300–400 nm) with a 10 nm wavelength step and 5 s light and 10 s dark cycles. PEC sensing experiments were performed using an amperometric method with an applied polarization potential of 0.2, 0.5, or 1.0 V vs. SCE. A defined quantity of glucose was added every 30 s after a stable photocurrent was observed in the system. Interfering tests were performed in solutions with the addition of 47 µmol dm^−3^ of interfering component (urea, uric acid, and lactic acid). An intravenous glucose solution (50 mg mL^−1^, Freeflex) was used for real-life sample analyses. The repeatability of the ATO PEC sensor was studied at one and three months after the first usage, and the experiments were preceded by a one-hour exposure of the photoanode (placed in the electrolyte) to UV light (350 nm).

## 3. Results

### 3.1. Photoelectrochemical Properties of ATO

**Figure 1:**
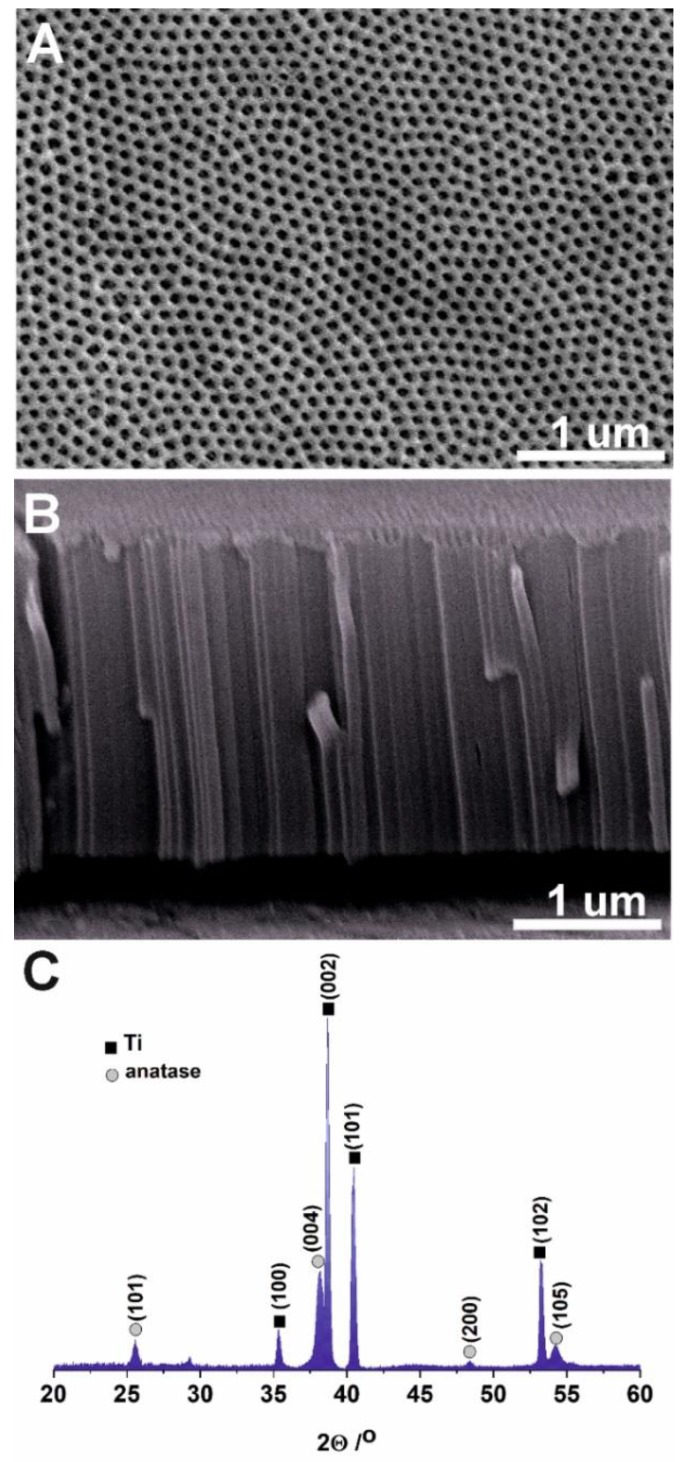
Scanning electron micrographs of top-view (**A**) and the corresponding cross-sectional view (**B**) of ATO. XRD pattern of ATO annealed at 400 °C for 2 h in air (**C**).

ATO is an attractive material for PEC applications due to its unique morphology, as presented in the scanning micrographs in Figure 1. As can be seen, the top ATO layer consists of pores with an average diameter of 65 ± 7 nm (Figure 1A), and the thickness of anodic oxide layers obtained at potential of 40 V was about 2.2 ± 0.3 µm (Figure 1B). As-received amorphous ATO layers have a porosity of about 30% [38]. It is widely recognized that the heat-treatment of ATO layers at 400 °C does not affect the morphological features [35]. Conducted XRD analysis of ATO and spectrum obtained is shown in Figure 1C. In this figure, predominant peaks are observed at 2Θ of 25.5°, 38.0°, and 48.3°. By comparing these peaks to reported by Jarosz et al., and using the ICDD database, they were assigned as the corresponding anatase crystalline structure of plane (101), (004), and (200).

**Figure 2:**
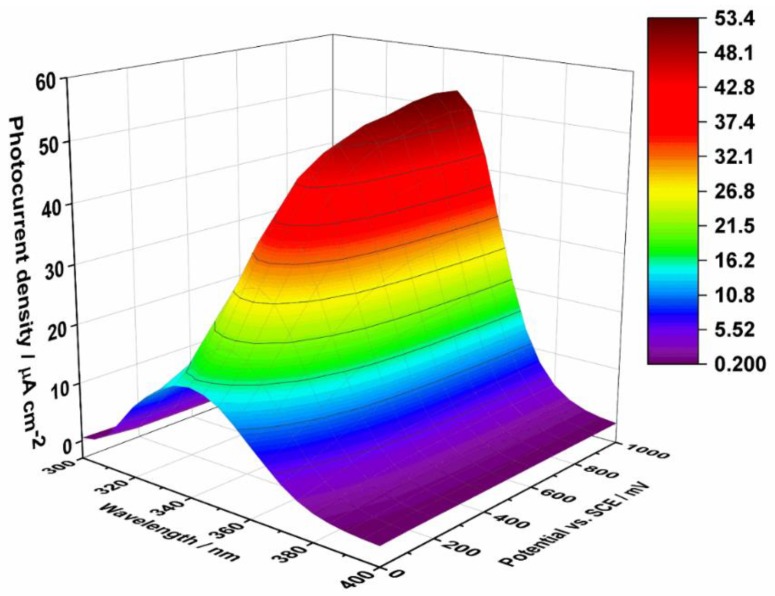
Photocurrent density as a function of incident light wavelength and applied potential recorded in 0.1 M KNO_3_ for the ATO layers annealed at 400 °C.

Figure 2 presents PEC activity measured as a steady-state current density in 0.1 M KNO_3_ at the ATO electrode, which was polarized over the potential range of 0–1 V vs. SCE. As can be seen, ATO is able to generate anodic photocurrent in the wavelength range of 300–400 nm, and the highest values are observed at 350 nm. Based on these results, 350 nm was chosen as the optimal irradiation wavelength for the subsequent amperometric sensing of glucose in our PEC system.

**Figure 3:**
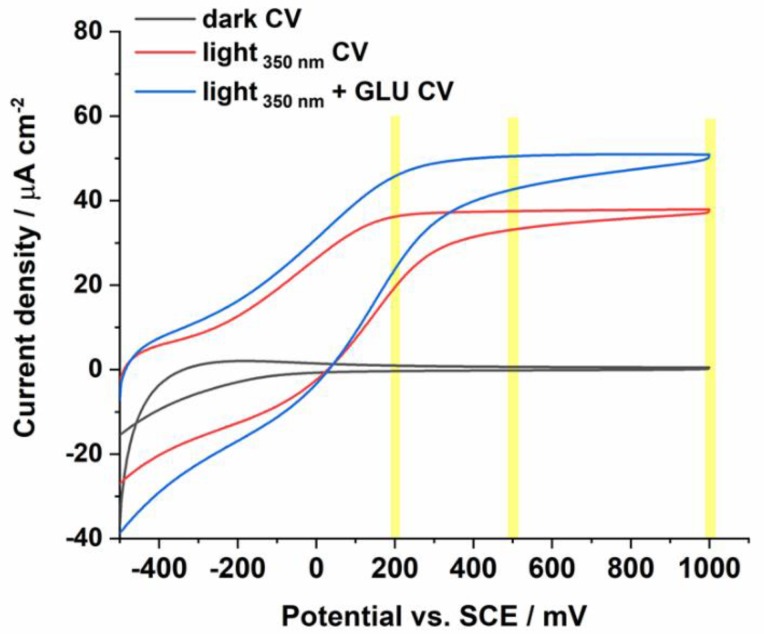
Cyclic voltamperograms recorded in 0.1 M KNO_3_ in the dark, and during irradiation with a wavelength of 350 nm in the absence or presence of glucose.

Cyclic voltamperograms of glucose, and a blank solution were recorded at the ATO electrode in the dark and during illumination with light of wavelength 350 nm (Figure 3). As expected, as a result of radiation, much higher current densities are observed than were measured in the dark, which increased even more after the addition of glucose. This indicates that glucose oxidation occurs in the range of applied potentials from about 0.2 V to 1.0 V vs. SCE. The polarization potentials of 0.2, 0.5, and 1 V vs. SCE (marked in yellow) were selected for further studies due to the observed steady-state photocurrent which allow determination of glucose using a chronoamperometric technique.

### 3.2. PEC Sensing of Glucose

**Figure 4:**
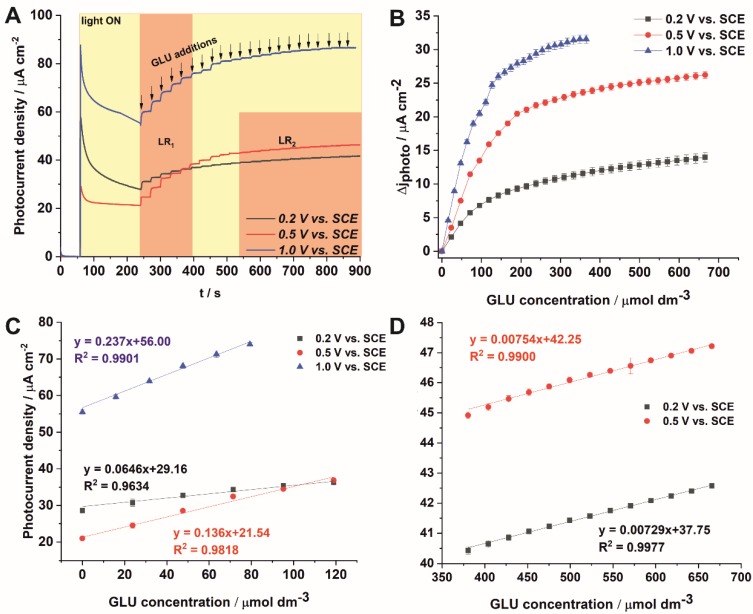
Amperometric response of ATO layers successive addition of GLU at different applied potentials (**A**). Relative photocurrent density values as a function of GLU concentration at different applied potentials (**B**). Corresponding calibration curves for the lower LR1 (**C**) and higher linear range LR2 (**D**) of GLU concentrations.

The typical amperograms obtained by successive additions of GLU at intervals for tested conditions are depicted in Figure 4A, where LR stands for a linear response region. As can be seen, at continuous light illumination each glucose addition causes an increase in the generated photocurrent for all photoelectrochemical systems. At low polarization potentials (0.2 and 0.5 vs. SCE) two linear regions are observed in the sensor response, namely LR1 and LR2 for the glucose concentration of 0–119 µmol dm^−3^ and 380–665 µmol dm^−3^, respectively. For the polarization potential of 1 V vs. SCE, the system displays a linear region in a range of concentrations between 0 and 79 µmol dm^−3^. The difference between the photocurrent density after the addition of glucose, and the dark current as a function of glucose concentration is shown in Figure 4B. For polarization potentials lower than 1 V vs. SCE a similar behavior is observed, i.e., initial large changes in the relative photocurrent density of the system with increasing glucose concentration to about 120 µmol dm^-3^, followed by smaller photocurrent density changes with increasing glucose concentration. The asymptotic behavior in the Figure 4B corresponds to a saturation effect that is probably related to the exhaustion of active centers at the electrode surface. At the polarization potential of 1 V vs. SCE, the saturation effect is observed earlier, for lower glucose concentrations. After reaching the concentration of 300 µmol dm^−3^, the PEC response does not change significantly. This result may encourage reaching a maximal oxidation capacity of the photoanode at those conditions (mass transport of GLU is no longer a limiting step) [39]. The calibration plots for lower (LR1) and higher (LR2) glucose concentration ranges are shown in Figure 4C,D, respectively. In contrast to Feng et al. [13], it was observed that the increase in applied polarization potential (for the LR1) leads to improvement of the sensitivity of the photoelectroanalytical method. The limit of detection (LOD) obtained for the potential of 0.5 V vs. SCE was lower (7.8 mM) than the values found for the potential of 0.2 and 1 V vs. SCE (15.6 and 16.3 mM, respectively). For the LR2 concentration range, the sensitivities are significantly lower and LOD values much higher. Namely, the sensitivity and LOD values of the electrode were found to be 7.3 µA mmol^−1^ cm^−2^, and LOD = 1 M for 0.2 V vs. SCE, and 7.5 µA mmol^−1^ cm^−2^ and LOD = 0.5 M for 0.5 V vs. SCE.

A comparison of our PEC sensor with some of the other reported in the literature non-enzymatic glucose sensors based on TiO_2_ nanotubes revealed that our new sensor exhibits a good performance characterized by high sensitivity, and a considerable wide linear range. Moreover, the sensitivity of the proposed PEC sensor is higher than some electrochemical sensors based on TiO_2_ nanotubes, such as helical TiO_2_ nanotubes modified with Cu_2_O (14.5 µA mM^−1^ cm^−2^) [29], CuO/TiO_2_ arrays (79.8 µA mM^−1^ cm^−2^) [30], Pt/TiO_2_ nanotubes (63.8 µA mM^−1^ cm^−2^) [23], and AgNPs deposited on TiO_2_ nanotubes (3.7 µA mM^−1^ cm^−2^) [19]. However, some electrochemical sensors have better detection limits. As already mentioned, PEC sensors are characterized by a fast response time to the addition of the analyte.

**Figure 5:**
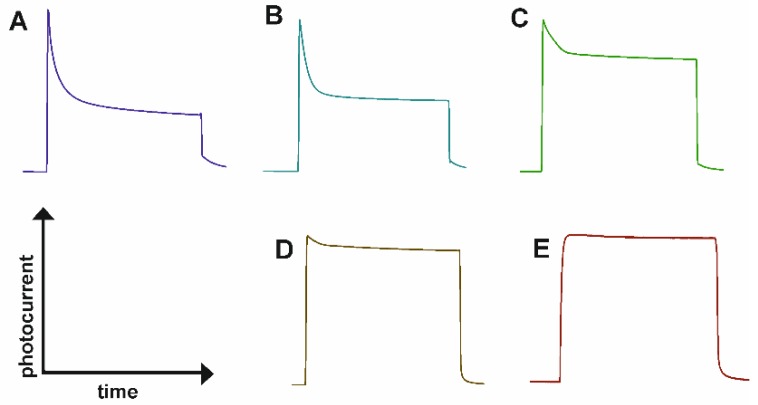
Photocurrent transient observed for a electrode potential of 0 V (**A**), 0.2 V (**B**), 0.5 V (**C**), 0.7 V (**D**) and 1.0 V (**E**) vs. SCE during exposure to light with a wavelength of 350 nm measured for ATO in 0.1 M KNO_3_.

Due to the different kinetics of steady-state photocurrent generation, the response time of the PEC sensor at different polarization potentials may be diverse. Figure 5 shows photocurrent transients, observed for a few electrode potentials during exposure to light with a wavelength of 350 nm.

**Figure 6:**
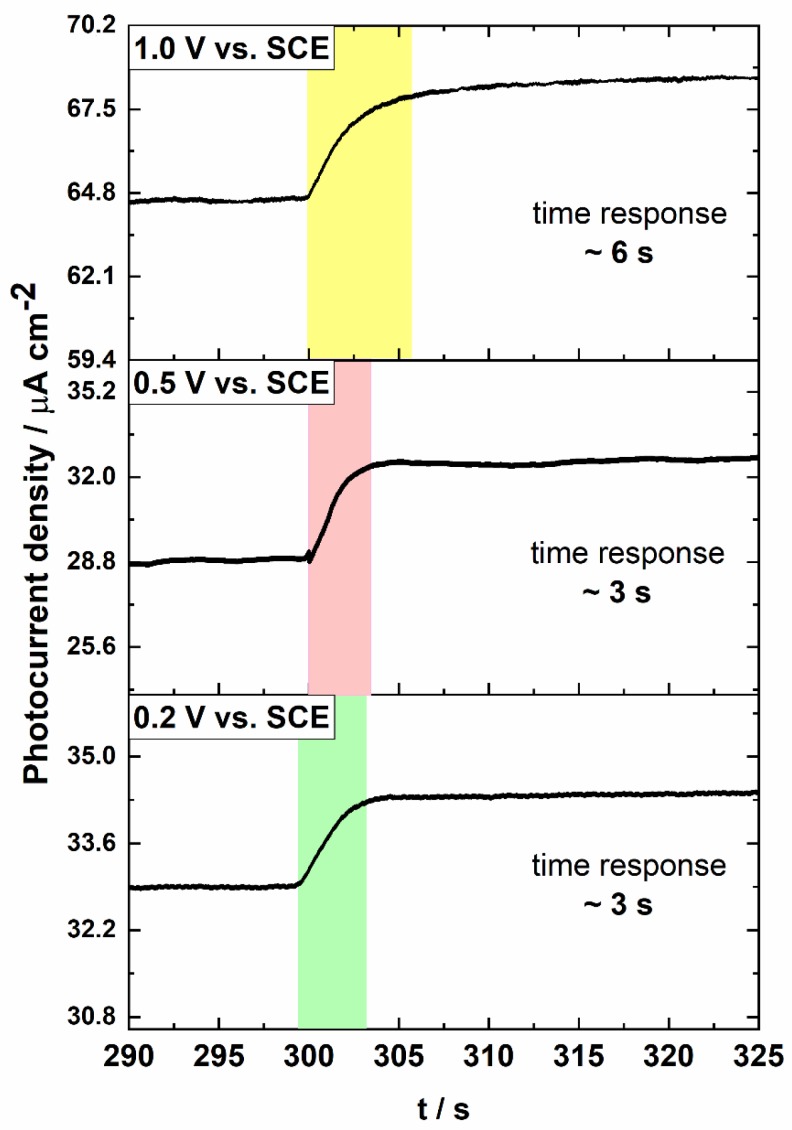
Average time response of the PEC sensor based on the ATO layer for different polarization potentials.

As can be seen in Figure 5, a voltage dependent charge recombination is clearly observed. The results indicate that increasing polarization potential decreases surface recombination rate. A recombination process may occur either from the surface charge or via the trap states [40]. When a low potential (0 V vs. SCE) is applied, the photocurrent density exhibits a sharp anodic peak occurring immediately after exposure to irradiation, and an exponential decay which eventually reaches a new steady-state photocurrent. This anodic spike is related with accumulation of charge near the surface of semiconductor [40,41,42]. On the other hand, photogenerated electrons can be extracted by the external circuit faster or slower. In other words, transport to back contact occurs with a different extraction time depending on the applied potential. Upon increasing the polarization potential of the photoanode, charge extraction kinetics is slower. The process is related to an increased number of electron traps that are empty under increasing bias. This decay (which indicates a recombination rate) is becoming smaller with increasing applied potential up to 1.0 V vs. SCE, at which stage the onset potential is significantly smaller than the applied potential, and the steady-state photocurrent is reached almost immediately [40,41]. Therefore, at the lower polarization potentials (0.2, 0.5 V vs. SCE) the electrode reacts faster (~3 s) to the glucose addition. This is supported by the results in Figure 6 that show the average transient response of the ATO layer-based PEC sensor at different polarization potential. For the highest applied potential (1 V vs. SCE) at which the steady-state photocurrent is observed, response time is longer (6 s) due to a lower mobility of charge carriers. This aspect has not been sufficiently studied before, the published papers on PEC sensors based on unmodified anodic titanium oxide report a relatively long response time of sensors e.g., 20 s [17] and 56 s [18]. 

### 3.3. Interfering Substances

**Figure 7:**
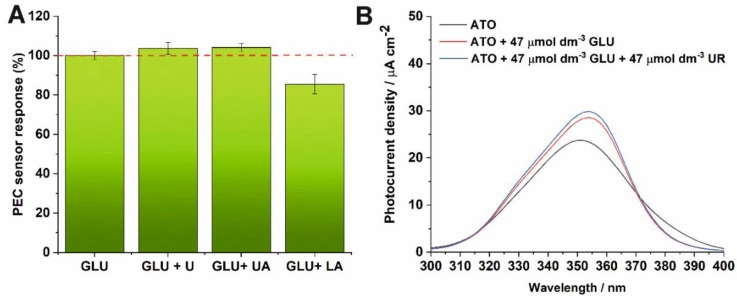
(**A**) Influence of interfering substances on photocurrent densities in the presence of glucose at 350 nm. (**B**) Photocurrent density of the ATO photoanode measured in 0.1 M KNO_3_ at the applied potential of 0.5 V vs. SCE as a function of incident light wavelength in the presence of glucose and urea.

As was mentioned before, the electrochemical determination of glucose may be overestimated or underestimated by additional electroactive compounds present in the analyzed sample. Selectivity of the proposed PEC sensor towards GLU oxidation was accessed by measuring the generated photocurrent in the presence of other some electroactive substances. The chronoamperometric measurements were carried out at 0.5 V vs. SCE under UV light illumination. The influence of adding 47 µmol dm^−3^ of interfering species commonly present in physiological samples, such as urea (U), uric acid (UA), and lactic acid (LA) on photocurrent response was tested with the same GLU concentration. The percentage of the PEC sensor response to GLU in the presence of each of these interfering species is shown in Figure 7A. A 13% decrease in the photocurrent response for GLU oxidation was only observed in the presence of lactic acid in 0.1 M KNO_3_. An ~4% increase in the PEC response was observed in the presence of urea and uric acid in 0.1 M KNO_3_.

In addition, we have also studied whether glucose can be oxidized over the whole wavelength range that ATO absorbs radiation, and whether the presence of interferers (e.g., uric acid) affects the observed PEC response. The results are shown in Figure 7B. As can be seen, the generated photocurrent decreases in the presence of glucose and UA only for wavelengths from 375 nm to 400 nm. However, glucose can be oxidized in the wavelength range of 300–375 nm, where enhancement in the generated photocurrent is observed. 

### 3.4. Real-Life Samples Analysis

**Figure 8:**
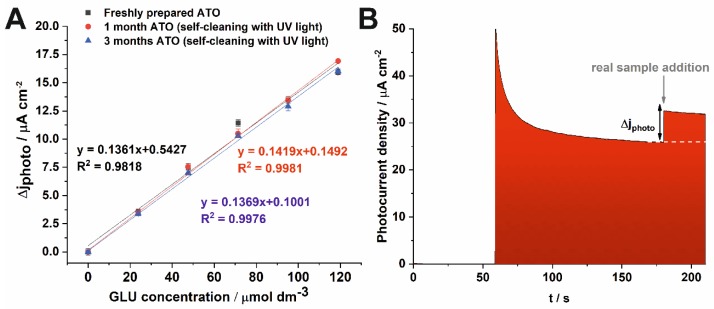
Calibration plots for the lower LR1 concentration range obtained at 0.5 V vs. SCE for the freshly prepared ATO electrode and after 1 month of its storage (**A**). Photocurrent density of the ATO photoanode measured in 0.1 M KNO_3_ at the potential of 0.5 V vs. SCE in the presence of real sample addition (**B**).

To demonstrate the applicability of the proposed ATO PEC sensor to real-life samples analysis, the determination of glucose in a glucose injection solution (Freeflex) was performed. The analyses were made with a freshly prepared ATO sensor, and after its storage for one and three months in air at ambient conditions. Before re-using of the ATO sensor, a self-cleaning procedure (see Introduction) based on UV light illumination of the photoanode immersed in water at 30 °C for 1 h was employed. The calibration plots obtained for measurements before, and after storage are very similar (Figure 8A), however after one month of storage and self-cleaning a slightly better sensitivity (0.142 µA µmol^−1^ cm^−2^) compared to as-received photoanode was obtained. This demonstrates that the photoanode was not poisoned or blocked by oxidation products or contaminants, and can be used repeatedly (even after a relatively long term storage) for the detection of glucose. 

The determination of glucose in the real-life sample was performed in a similar way to measurements for calibration tests (Figure 8B).

The results obtained from seven independent measurements (Table 2) show a very good agreement between the concentration of glucose determined by using the proposed sensor and that declared by the manufacturer of the injection sample. The satisfactory recovery of the experimental results ranging from 90% to 93% were found. A good repeatability of the method is demonstrated by the mean relative standard deviation (RSD) values lower than 3%. 

## 4. Conclusions

In this paper, we present a PEC sensor based on non-modified ATO with designed morphology that provides better PEC sensing properties than the ones shown previously. The ATO sensor operates at two different potentials with a good sensitivity and fast response time (3 s). The highest sensitivity of 0.237 µA µmol^−1^ cm^−2^ (237 µA mmol^−1^ cm^−2^) was found for the bias of 1 V vs. SCE. For the potential of 0.5 and 0.2 V vs. SCE, the sensitivity of 0.136 and 0.065 µA µmol^−1^ cm^−2^ was observed, respectively. The limit of detection (LOD) was obtained for the potential of 0.5 V vs. SCE (7.8 mM). The effect of interfering substances was studied at the applied potential of 0.5 V vs. SCE. It was found that urea and uric acid affect PEC response in a small extent (less than 4%). A long-term stable PEC sensor without any sensitivity loss was obtained by exploitation of self-cleaning properties of TiO_2_ under UV illumination. It was shown that the sensor is stable up to 3 months with a satisfactory recovery of results (90–93%). The proposed sensor can be used for determination of glucose in real-life samples. In addition, glucose oxidation occurs almost in the entire wavelengths appropriate for photoactivity of the given semiconductor material. In further work, we will focus on shifting the absorption of ATO towards visible light in order to fabricate a PEC sensor self-powered by solar radiation.

## Figures and Tables

**Table 1:** Photoelectrochemical sensors based on anodic TiO_2_ for glucose determination (where: NPs: nanoparticles, NT: nanotubes, LOD: limit of detection).

Electrode	Applied Potential	Sensitivity [µA µmol^−1^ cm^−2^]	Linear Range [µmol dm^−3^]	LOD [µmol]	Response Time [s]	Reference
Anodic TiO_2_ NT GLU	0.2 vs. Ag/AgCl (tested range: 0.2–1.0 V)	0.14	10–1200	2.7	~56 s	[18]
Anodic TiO_2_ NT GLU	0.2 V vs. Ag/AgCl	-	-	-	-	[14]
Anodic TiO_2_ NT GLU	0.2 V vs. Ag/AgCl	0.12	0–1000	6.49	~20 s	[17]
Anodic TiO_2_ NT + AgNPs GLU		0.194	0–700	0.53		
Anodic TiO_2_ NT + PtNPs GLU		0.076	0–650	13.5		

**Table 2:** The application of PEC sensor based on the ATO layer for determination of glucose in the real sample.

	Δj_photo_ (µA cm^−2^)	GLU Concentration (µmol dm^−3^)	Recovery (%)	Repeatability (% RSD)
Freeflex added	-	47.6	-	-
Freshly prepared ATO	6.49	43.7 ± 1.3	92	2.97
1 month storage Self-cleaning with UV light	6.25	43.0 ± 0.9	90	2.10
3 month storage Self-cleaning with UV light	6.16	44.3 ± 0.8	93	2.82
